# Exploring the Key Signaling Pathways and ncRNAs in Colorectal Cancer

**DOI:** 10.3390/ijms25084548

**Published:** 2024-04-21

**Authors:** Yun Ju Lee, Woo Ryung Kim, Eun Gyung Park, Du Hyeong Lee, Jung-min Kim, Hae Jin Shin, Hyeon-su Jeong, Hyun-Young Roh, Heui-Soo Kim

**Affiliations:** 1 Department of Integrated Biological Sciences, Pusan National University, Busan 46241, Republic of Korea; lsg5821@naver.com (Y.J.L.); dnfud647@pusan.ac.kr (W.R.K.); ehdtodt@pusan.ac.kr (E.G.P.); doo2080@naver.com (D.H.L.); jmk95@naver.com (J.-m.K.); 0705haejin@naver.com (H.J.S.); tbd97@pusan.ac.kr (H.-s.J.); 2 Institute of Systems Biology, Pusan National University, Busan 46241, Republic of Korea; susan9416@naver.com; 3 Department of Biological Sciences, College of Natural Sciences, Pusan National University, Busan 46241, Republic of Korea

**Keywords:** colorectal cancer, Wnt signaling pathway, PI3K/AKT/mTOR signaling pathway, MAPK signaling pathway, TGF-β signaling pathway, p53 signaling pathway, miRNA, long non-coding RNA, circular RNA, CeRNA

## Abstract

Colorectal cancer (CRC) is the third most prevalent cancer to be diagnosed, and it has a substantial mortality rate. Despite numerous studies being conducted on CRC, it remains a significant health concern. The disease-free survival rates notably decrease as CRC progresses, emphasizing the urgency for effective diagnostic and therapeutic approaches. CRC development is caused by environmental factors, which mostly lead to the disruption of signaling pathways. Among these pathways, the Wingless/Integrated (Wnt) signaling pathway, Phosphatidylinositol 3-kinase/protein kinase B/mammalian target of rapamycin (PI3K/AKT/mTOR) signaling pathway, Mitogen-Activated Protein Kinase (MAPK) signaling pathway, Transforming Growth Factor-β (TGF-β) signaling pathway, and p53 signaling pathway are considered to be important. These signaling pathways are also regulated by non-coding RNAs (ncRNAs), including microRNAs (miRNAs), long non-coding RNAs (lncRNAs), and circular RNAs (circRNAs). They have emerged as crucial regulators of gene expression in CRC by changing their expression levels. The altered expression patterns of these ncRNAs have been implicated in CRC progression and development, suggesting their potential as diagnostic and therapeutic targets. This review provides an overview of the five key signaling pathways and regulation of ncRNAs involved in CRC pathogenesis that are studied to identify promising avenues for diagnosis and treatment strategies.

## 1. Introduction

Colorectal cancer (CRC) is the third most commonly diagnosed cancer in both males and females, with an estimated 81,540 new cases in males and 71,720 new cases in females in 2024, according to the American Cancer Society [1]. In addition, CRC is estimated to cause significant mortality, with approximately 28,700 deaths in males, ranking the top three among males, and 24,310 deaths in females, positioning it as the fourth among females. An examination of the five-year disease-free survival rate revealed a notable trend: as the stage of CRC advances, the survival rates decrease. Specifically, the five-year disease-free survival rates are 91.0% for stage I, 79.8% for stage II, 63.3% for stage III, and 18.9% for stage IV [2]. These statistics highlight the severity of this disease and underscore the need for effective strategies for diagnosing and treating CRC.

CRC develops as a result of abnormal growth cells in the colon or rectum, which over time turn cancerous [3]. It is commonly known that obesity, overweight, a lack of physical activity, smoking, alcohol consumption, and diet are risk factors for CRC [4,5,6]. Most CRCs are thought to be caused by genetic alterations induced by these environmental factors [7]. Consequently, the genetic alterations can induce the aberrant activation of signaling pathways, leading to tumor progression [8]. Many dysregulated signaling pathways in CRC have been identified, most notably the Wingless/Integrated (Wnt) signaling pathway, Phosphatidylinositol 3-kinase/protein kinase B/mammalian target of rapamycin (PI3K/AKT/mTOR) signaling pathway, Mitogen-Activated Protein Kinase (MAPK) signaling pathway, Transforming Growth Factor-β (TGF-β) signaling pathway, and p53 signaling pathway [9]. Aberrant signaling pathways occur due to abnormalities in the expression of genes that function in the pathway, which also affects genes downstream [8]. As these studies continue to expand, there is increasing interest in non-coding RNAs (ncRNAs) as regulators of gene expression.

Unlike coding RNAs, ncRNAs, which are transcribed from DNA but not translated into protein, have been shown to act as a regulator [10]. Well-known ncRNAs such as microRNAs (miRNAs), long non-coding RNAs (lncRNAs), and circular RNAs (circRNAs) have been extensively studied and shown to directly or indirectly regulate the expression of genes involved in CRC, thereby influencing various biological processes [11]. Due to their altered expression patterns and their significant function in CRC, these ncRNAs can also be used not only for the diagnosis but also as potential targets for the treatment. In this review, we aim to summarize the five major signaling pathways and ncRNAs implicated in CRC, to identify potential targets for diagnosis and treatment.

## 2. Multiple Major Signaling Pathways in CRC

**Figure 1:**
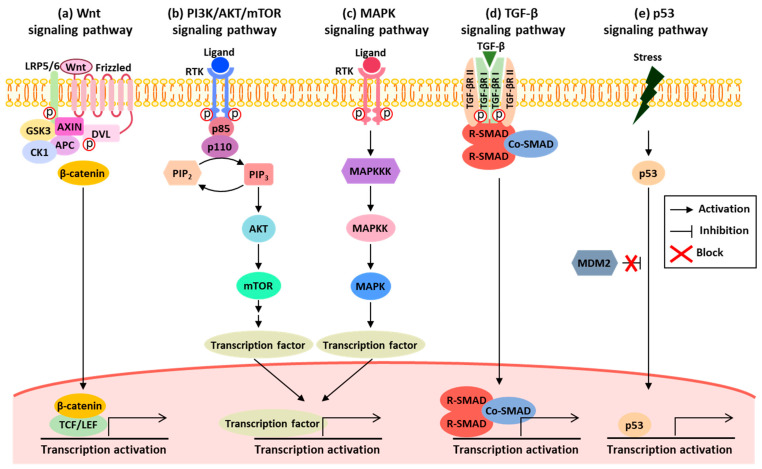
Schematic diagram of activated signaling pathways: (**a**) the Wnt signaling pathway is activated in the presence of Wnt. When Wnt binds to the Frizzled receptor, it dimerizes with LRP5/6, leading to the recruitment of the Axin/GSK3/APC/CK1 complex to the cell membrane. This results in the translocation of β-catenin from the cytoplasm to the nucleus. In the nucleus, β-catenin interacts with TCF/LEF transcription factors, thereby inducing the expression of downstream target genes. (**b**) The PI3K/AKT/mTOR signaling pathway is activated when a ligand binds to receptors such as RTK. Activated PI3K phosphorylates PIP2 to convert it to PIP3. Then, PIP3 activates downstream genes, such as AKT and mTOR. (**c**) The MAPK signaling pathway is initiated when a ligand binds to RTK, leading to the phosphorylation and activation of MAP3K. Activated MAP3K phosphorylates the downstream MAP2K, which in turn phosphorylates MAPK. This phosphorylation cascade transmits signals from the cell surface receptor to the nucleus, influencing gene expression by activating various kinases, enzymes, and transcription factors downstream. (**d**) The TGF-β signaling pathway is activated when TGF-β directly or indirectly binds to TGF-βR II. TGF-βR II recruits and phosphorylates TGF-βR I, which, in turn, phosphorylates R-SAMD. The phosphorylated R-SMAD then forms a trimeric complex with Co-SMAD. This complex translocates to the nucleus, where it acts as a transcription factor, influencing gene expression. (**e**) The p53 signaling pathway is initiated when p53 remains stabilized due to its failure to bind with MDM2, an E3 ubiquitin ligase. This stabilization of p53 leads to the induction of downstream gene expression, resulting in the activation of the pathway. Wnt signaling pathway: Wingless/Integrated signaling pathway; PI3K/AKT/mTOR signaling pathway: Phosphatidylinositol 3-kinase/protein kinase B/mammalian target of rapamycin signaling pathway; RTK: receptor tyrosine kinase; PIP2: phosphatidylinositol 4,5-bisphosphate; PIP3: phosphatidylinositol 3,4,5-trisphosphate; MAPK: Mitogen-Activated Protein Kinase; MAP3K: MAP kinase kinase kinase; MAP2K: MAP kinase kinase; TGF-β: Transforming Growth Factor-β; TGF-βR: Transforming Growth Factor-β receptor; R-SAMD: receptor-regulated SMAD; Co-SMAD: common-mediator SMAD; MDM2: mouse double minute 2 homolog.

In the human body, various signaling pathways exist, regulating essential cellular processes. However, when these pathways are disrupted, they can lead to various diseases [12]. CRC is no exception, and through numerous studies, differences in signaling pathways between normal and CRC patients have been extensively identified [8]. By comparing these signaling pathways with KEGG pathways (map05210), five signaling pathways play crucial roles in influencing CRC: the Wnt signaling pathway, PI3K/AKT/mTOR signaling pathway, MAPK signaling pathway, TGF-β signaling pathway, and p53 signaling pathway [9,13]. Although these signaling pathways ultimately regulate similar cellular characteristics, there are differences in the genes involved and the factors that trigger the activation of each gene within these pathways (Figure 1). Therefore, in this section, we focus on the changes in gene expression associated with each of these signaling pathways and discuss how these factors affect CRC.

### 2.1. Wnt Signaling Pathway

The complex and highly conserved Wnt signaling pathway is essential for embryonic development, tissue homeostasis, and various physiological processes [14,15,16]. The presence or absence of Wnt, a ligand, influences the expression levels of genes, particularly the transcriptional co-activator β-catenin, associated with the Wnt signaling pathway. In the absence of Wnt, β-catenin is phosphorylated by the Axin/GSK3/APC/CK1 complex and degraded by the ubiquitin-dependent proteasome, maintaining it at a low level [17]. However, in the presence of Wnt, Wnt binds to the Frizzled receptor, activating the Wnt signaling pathway [14,18,19]. The Frizzled receptor dimerizes with LRP5/6 and recruits the Axin/GSK3/APC/CK1 complex to the cell membrane, preventing the phosphorylation and degradation of β-catenin. Accumulated cytoplasmic β-catenin then translocates to the nucleus, where it interacts with TCF/LEF transcription factors, inducing the expression of Wnt target genes.

The Wnt signaling pathway has been extensively studied to prove its critical role in CRC, and the hyperactivation of this signaling pathway has been observed from early to advanced stages of CRC [20,21,22]. In this signaling pathway, β-catenin and adenomatous polyposis coli (APC) especially have been identified as representative core genes with frequent genetic changes in CRC, which have been found to significantly impact the development and progression of the disease [23,24,25]. According to a study, it was confirmed that β-catenin was activated by Wnt1, which plays an important role in CRC progression, and that a high expression of nuclear β-catenin was associated with poor prognosis [26]. Similarly, it was revealed that the activation of β-catenin in CRC contributed to the aggressiveness of CRC by manipulating cell-cell junctions and inducing epithelial-mesenchymal transition (EMT) [27]. In a different study, it was found that β-catenin was activated by HMGB3, leading to the activation of downstream components of the Wnt signaling pathway, including c-myc and MMP7 [28]. This activation promoted cell growth and migration, influencing the carcinogenesis and development of CRC. Unlike β-catenin, APC is known to act as a negative regulator in the Wnt signaling pathway and is recognized as a tumor suppressor [29]. In one study, it was observed that the overexpression of SMYD2, a negative regulator of APC2, was associated with poor prognosis [30]. The knockdown of SMYD2 led to a decrease in cell proliferation, migration, and invasion, along with an upregulation of APC2 and a downregulation of the expression of E-cadherin, inducing EMT. Additionally, inhibiting SMYD2 reduced lung metastasis, confirming that SMYD2 plays an important role in CRC by regulating the expression of APC2 and influencing the Wnt signaling pathway.

### 2.2. PI3K/AKT/mTOR Signaling Pathway

The PI3K/AKT/mTOR signaling pathway is an important intracellular signaling cascade that is essential for controlling many cellular functions, including proliferation, survival, DNA repair, apoptosis, and gene transcription [12,31,32]. PI3K is known as a conserved lipid kinase family, divided into three classes based on their structure and specific substrates. While classes II and III remain relatively less-explored areas, class I has been extensively researched. Class I PI3K can be further subdivided into classes IA and IB, depending on the types of catalytic and regulatory subunits. Class IA consists of catalytic subunits, namely p110 (p110α, p110β, and p110 δ), and regulatory subunits p85 (p85 α, p55α, p50α, p85β, and p55γ) [33,34,35]. On the other hand, class IB PI3K is composed of the catalytic subunit p110γ and the regulatory subunit p101. PI3K can be activated downstream of numerous growth factors, including the fibroblast growth factor, vascular endothelial growth factor, and receptors, such as receptor tyrosine kinases [36]. Activated PI3K phosphorylates phosphatidylinositol 4,5-bisphosphate to convert it into phosphatidylinositol 3,4,5-trisphosphate; subsequently, phosphatidylinositol 3,4,5-trisphosphate activates AKT [33,36]. Activated AKT induces the phosphorylation of other substrates in both the cytosol and nucleus, particularly promoting the activation of mTOR, thereby activating the PI3K/AKT/mTOR signaling pathway. In particular, this signaling pathway plays an important role in the early and late stages of CRC, and dysregulation of this signaling pathway leads to carcinogenesis by altering proliferation, angiogenesis, and apoptosis [37,38,39].

One study has been conducted to investigate the correlation between the expression of PI3K and primary versus metastatic CRC [40]. It was found that metastatic lesions in CRC patients exhibited a higher expression of PI3K compared to primary lesions, suggesting a potential association with metastasis. To examine the actual impact of PI3K on CRC, the PI3K inhibitor LY294002 was used and resulted in the induction of apoptosis in CRC cell lines, indicating the functional significance of PI3K. According to another study, the overexpression of AKT in CRC cell lines was shown to increase their proliferative, migratory, and invasive capacities [41]. Moreover, in a xenograft mouse model of CRC, tumors overexpressing AKT exhibited rapid growth, increased vascularization, and the induction of expression of EMT markers. In a different study, it was observed that a higher expression of mTOR in CRC patients correlated with decreased overall survival and disease-free survival probabilities [42]. Additionally, the treatment of CRC cell lines with mTOR inhibitors, rapamycin, and PP242 resulted in a significant decrease in the number and diameter of spheroid cell models derived from CRC cells.

Besides the mentioned studies, there is extensive research on how changes in the expression of genes involved in the PI3K/AKT/mTOR signaling pathway impact CRC. In a study, the artificial overexpression of LACTB, which is downregulated in both CRC patients and cell lines, led to a decrease in PIK3R3 expression and a reduction in the expression of proteins associated with the PI3K/AKT/mTOR signaling pathway [43]. Furthermore, downregulated PIK3R3 increased autophagy and decreased the proliferation, EMT, and tumor size in CRC cell lines or mouse models. Taken together, these results suggest that LACTB influences CRC development by regulating the PI3K/AKT/mTOR signaling pathway. In another study, it was observed that TGM3 was downregulated in CRC patients and cell lines [44]. Increasing the expression of this gene was found to decrease the phosphorylation of PI3K and AKT, indicating the inactivation of the PI3K/AKT signaling pathway suppressed the proliferation and metastasis of CRC cells. Similarly, it has been revealed that MUC3A, which is overexpressed in CRC and associated with poor prognosis, activates the PI3K/AKT/mTOR signaling pathway [45]. The knockout of MUC3A resulted in the inhibition of the PI3K/AKT/mTOR signaling pathway and induced proliferation, cell cycle arrest, and the invasion of CRC cells.

### 2.3. MAPK Signaling Pathway

The MAPK signaling pathway is an essential intracellular signaling system that controls multiple cellular processes, such as cell proliferation, differentiation, survival, and apoptosis [46,47]. This signaling pathway is initiated when a ligand binds to receptor tyrosine kinases, leading to the phosphorylation and activation of the MAPK kinase kinase (MAP3K) [48,49]. Activated MAP3K phosphorylates MAPK kinase (MAP2K), which, in turn, phosphorylates MAPK, activating various kinases, enzymes, and transcription factors, thereby transmitting signals from cell surface receptors to the nucleus and impacting gene expression.

In the case of MAPK, it consists of three main families: extracellular signal-regulated kinase 1/2 (ERK1/2), c-Jun N-terminal kinase (JNK), and p38. These families are identified to play important roles in the development, progression, and oncogenic impact of CRC, acting individually or concurrently within the specific cellular context and signaling requirements in CRC [5,50]. There are several studies on the independent action of MAPK in CRC. One study revealed the influence of the ERK/MAPK signaling pathway on the progression of CRC by demonstrating a decrease in CRC cell proliferation and migration when the MAP2K inhibitor U0126 was used to block the ERK/MAPK signaling pathway [51]. Similarly, another study revealed that the knockdown of COPB2, which was overexpressed in CRC, led to a reduction in the proliferation and induction of apoptosis in CRC cells through the JNK signaling pathway [52]. In a different study, activated FOXC1, whose degradation was inhibited by phosphorylation through p38, was overexpressed in CRC patients and correlated with poor prognosis [53]. The knockdown of FOXC1 induced a decreased migration and invasion of CRC cells. Additionally, it reduced the number of metastatic tumors in the CRC mouse model. 

In addition to cases where a single family independently affects the MAPK signaling pathway, it has been revealed that two MAPK families show combined actions within the signaling pathway. For instance, investigating both JNK and p38 MAPK signaling pathways simultaneously, it was found that S100A16 was overexpressed in CRC patients and correlated with poor prognosis [54]. The overexpression of S100A16 led to an increase in the expression of mesenchymal markers N-cadherin and vimentin, while decreasing the expression of the epithelial marker E-cadherin, implicating its involvement in EMT. Furthermore, treatment with the JNK inhibitor SP600125 and the p38 inhibitor SB203580 resulted in a decrease in cell migration and invasion. This suggests that S100A16 influences CRC through the JNK and p38 MAPK signaling pathways. Similarly, it has been confirmed that both ERK1/2 and p38 MAPK signaling pathways simultaneously influence CRC. The overexpression of CD24 induced the proliferation of CRC cells and increased the tumor size in a CRC mouse model, promoting tumorigenicity [55]. Additionally, increased CD24 promoted the activation of ERK1/2, Raf-1, and p38 MAPK, confirming that CD24-dependent ERK1/2 and p38 MAPK activation were necessary for CRC proliferation.

### 2.4. TGF-β Signaling Pathway

The TGF-β signaling pathway is a complex and multifunctional pathway that plays a crucial role in the regulation of cell growth, differentiation, immune responses, and homeostasis [56,57]. This pathway is activated by the binding of TGF-β (TGF-β I, TGF-β II, and TGF-β III) to its receptor, TGF-βR (TGF-βR I, TGF-βR II, and TGF-βR III). TGF-β I and TGF-β III bind directly to TGF-βR II, while TGF-β II can interact with TGF- βR II with the assistance of TGF-βR III [56,58,59,60]. Subsequently, TGF-βR II recruits and phosphorylates TGF-βR I for activation. Then, receptor-regulated SMADs (R-SMADs, SMAD 1, SMAD 2, SMAD 3, SMAD 5, and SMAD 8) are phosphorylated and activated to initiate the signaling cascade. In turn, the common-mediator SMAD (Co-SMAD, SMAD 4) interacts with R-SMAD to form a trimeric complex. This complex translocates into the nucleus and binds to DNA binding sites as transcription factors, fulfilling their role in gene expression. The activated TGF-β signaling pathway influences tumor progression and development in CRC [56].

A study confirmed that the expression of SMAD 1 was increased in CRC patient tissues [61]. When the expression of SMAD 1 was inhibited, the migration ability of CRC and the expression of N-cadherin were decreased, while the expression of E-cadherin was increased, indicating a reduction in EMT ability. Therefore, it can be anticipated that SMAD1 induces the migration of CRC cells and increases EMT in CRC patients. Another study confirmed the influence of ITGΒ5 on CRC by regulating the TGF-β signaling pathway [62]. ITGΒ5 was overexpressed in CRC patients, which correlated with poor survival. The knockdown of ITGB5 resulted in the decreased proliferation and invasion of CRC cells. Additionally, in a CRC mouse model, the suppression of ITGΒ5 led to reduced tumor size and metastasis. In addition, the repression of ITGΒ5 resulted in the decreased expression of TGF-βR I and phospho-SMAD 2, while increasing the expression of E-cadherin. This indicates that ITGΒ5, which was highly expressed in CRC, promotes the TGF-β signaling pathway and induces EMT in CRC. Similarly, it was revealed that the transcription activator EHF was overexpressed in CRC and correlated positively with poor prognosis [63]. EHF knockdown suppressed CRC cell proliferation, viability, and migration while inducing apoptosis. Moreover, in the CRC mouse model, repressed EHF resulted in reduced tumor size and metastasis to the liver. Further experiments revealed a positive correlation between the expressions of EHF and TGF-β1. It was demonstrated that EHF binds to the promoter of TGF-β1, thereby regulating its expression and implicating EHF in the tumorigenesis and metastasis of CRC.

### 2.5. p53 Signaling Pathway

The p53 signaling pathway is a crucial cellular pathway that plays a central role in regulating cellular responses to stress, including cell cycle, DNA repair, and apoptosis [64,65]. The p53 transcription factor is known to function as a tumor suppressor, aiding in the maintenance of genomic stability [6,66,67]. In unstressed cells, polyubiquitination by the E3 ubiquitin ligase mouse double minute 2 homolog (MDM2) leads to low levels of p53. MDM4 (MDMX), known as both a homology of MDM2 and a negative regulator of p53, interacts with MDM2 to bind to p53. As a result of this interaction, p53 is degraded via MDM2-dependent polyubiquitination, leading to the suppression of its transcriptional activity [6,68]. However, various stress signals, including DNA damage and abnormal growth signals, disrupt the interaction between p53 and MDM2. This disruption results in the stabilization of p53, enabling the activation of the transcription of target genes, which regulate various cellular responses, such as senescence and metabolism [69].

In CRC, mutations in the *TP53* gene, encoding p53, occur frequently, with approximately 43% of cases exhibiting mutations [6]. Mutations in p53 typically impair the function of wild-type p53, leading to a loss of its tumor suppressor properties. These alterations can affect cancer development and progression, such as cancer stem cell formation, cell proliferation, invasion, and metastasis [6,70]. One study found that CRC patients with *TP53* mutations with a complete loss of p53 had significantly different tumor sizes and decreased overall survival compared to CRC patients with wild-type *TP53* [71]. In another study, similar findings were observed where CRC patients with a positive p53 expression had higher overall survival compared to those with a negative p53 expression [72].

In addition to the direct impact of mutations in the *TP53* on CRC, it has been revealed that the expression level of other genes located upstream or downstream of the p53 affects CRC by influencing the p53 signaling pathway. One study confirmed that the p53 signaling pathway influenced cancer progression through the low expression of *MDM2*, a negative regulator of p53, in CRC patients with liver metastasis [73]. Similarly, an analysis using the GEO and GEPIA databases confirmed that *PRDX2* was overexpressed in CRC and correlated with poor prognosis [74]. The downregulation of PRDX2 led to reduced cell proliferation and tumor growth, accompanied by the upregulation of p53 expression. Furthermore, PRDX2 has been shown to interact with MDM2, leading to the increased ubiquitination and degradation of p53. Thus, PRDX2 is implicated in CRC progression by promoting the ubiquitinated degradation of p53 via the p53 signaling pathway. In another study, it was found that HAUS6 was overexpressed in CRC patients and was associated with shorter overall survival [75]. The knockdown of HAUS6 resulted in the activation of the p53 signaling pathway, leading to an increased expression of downstream p21. Consequently, HAUS6 induces tumor growth and proliferation by regulating the p53 signaling pathway. In addition, there is research indicating that GADD45B, overexpressed in CRC patient tissues, plays a role in apoptosis in CRC [76]. Additionally, while the anti-apoptotic member BCL-2 was upregulated, the pro-apoptotic member Bax was downregulated in these tissues. Furthermore, the knockdown of GADD45B disrupted the balance between anti-apoptotic and pro-apoptotic proteins, indicating that GADD45B influenced apoptosis through the p53 signaling pathway. In a different study, the knockdown of RPS15A, which was highly expressed in CRC patient tissues, has been shown to suppress cell proliferation and induce cell cycle arrest at the G0/G1 phase [77]. Additionally, the downregulation of RPS15A increased the expression of *p53* and *p21*, confirming that RPS15A induces CRC malignancy through the p53 signaling pathway.

## 3. ncRNA Regulation in CRC

**Figure 2:**
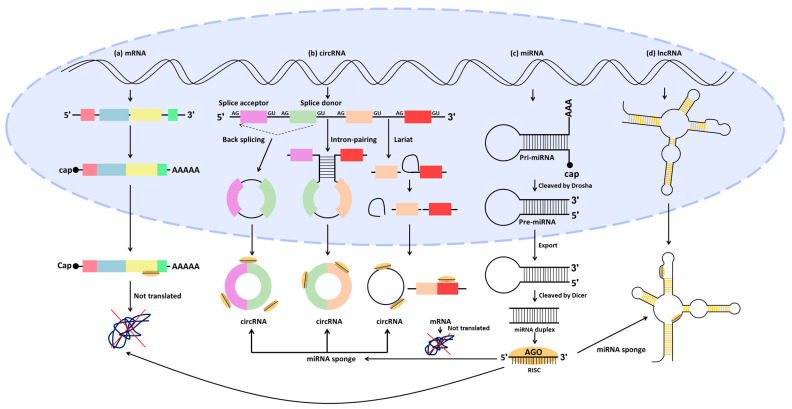
Biogenesis pathways and mutual regulatory relationships of mRNA, miRNA, lncRNA, and circRNA. (**a**) mRNA generated from a gene undergoes capping, tailing, and splicing to produce mature mRNA. This mature mRNA is regulated directly by miRNA or indirectly by lncRNA or circRNA in the cytoplasm, thereby controlling the translation of the mRNA. (**b**) CircRNA is generated through back-splicing, intron-pairing-driven circularization, and lariat-driven circularization. Back-splicing involves connecting the 5′ splice donor site downstream to the 3ʹ splice acceptor site upstream, forming a circular structure. In intron-pairing-driven circularization, circRNA is formed by the binding of inverted complementary sequences on the sides of long intron base pairs. Lariat-driven circularization occurs when the excision process of regular splicing fails, resulting in the generation of a circRNA. CircRNA binds to miRNA, indirectly influencing gene expression by preventing miRNA from binding to target genes. (**c**) Pri-miRNA is transcribed from the miRNA gene and is cleaved by Drosha to produce pre-miRNA. The pre-miRNA is then transported to the cytoplasm and processed to form a miRNA duplex by Dicer. One of the strands binds with AGO to form the RISC, which subsequently interacts with the target mRNA, leading to an inhibition of translation. (**d**) LncRNA is generated from DNA similarly to mRNA, undergoing capping, splicing, and tailing. Once generated, lncRNA in the cytoplasm binds to miRNA, indirectly affecting gene expression by inhibiting miRNA from binding to target genes. mRNA: messenger RNA; miRNA: microRNA; lncRNA: long non-coding RNA; circRNA: circular RNA; pri-miRNA: primary miRNA; pre-miRNA: precursor miRNA; AGO: Argonaute; RISC: RNA-induced silencing complex.

In the mid-1900s, non-protein-coding regions of the human genome were initially dismissed as junk DNA, with no apparent function unlike protein-coding genes [78]. However, with the completion of the Human Genome Project (HGP) in 2003, it became clear that protein-coding genes constitute only about 1.5% (21,000 genes) of the human genome, with non-protein-coding regions making up a significant proportion [79,80]. Subsequent projects, such as the Encyclopedia of DNA Elements (ENCODE) and the Functional Annotation of the Mammalian Genome (FANTOM), demonstrated that junk DNA contains essential regulatory elements for transcription, as well as sequences encoding ncRNAs involved in various biological mechanisms and functions [78,81]. Through these projects, sequencing techniques have significantly advanced and have been used to study ncRNAs in the human genome, revealing substantial insights [82]. ncRNAs, not translated to protein, are generally classified into two categories based on RNA transcript length: those shorter than 200 nucleotides are small ncRNAs, while longer ones are called lncRNAs [83]. miRNAs, representative small ncRNAs, and lncRNAs have been extensively studied in various diseases, including CRC, due to their direct and indirect regulation of protein-coding gene expression [84]. In recent years, circRNAs, ncRNA characterized by their circular structure, have gained attention and have been implicated in the regulation of gene expression, similar to lncRNAs [85]. Therefore, in this section, we aim to discuss the regulatory relationships of miRNAs, lncRNAs, and circRNAs, as well as their impact on CRC (Figure 2).

### 3.1. miRNA Regulation

miRNAs are a class of small RNA molecules that play a crucial role in the regulation of gene expression in various organisms [86]. They are typically short and single-stranded RNA molecules composed of approximately 18 to 24 nucleotides [87,88]. miRNAs regulate gene expression by binding to the miRNA response element (MRE) of messenger RNA (mRNA) transcribed from genes, thereby modulating post-transcriptional processes [89,90]. By regulating the levels of proteins produced by specific genes, miRNAs play important roles in various biological processes, including development, cell proliferation, differentiation, apoptosis, and immune response [91,92]. The biogenesis of mature miRNAs involves a series of steps (Figure 2). Initially, long primary miRNA transcripts (pri-miRNAs) are transcribed by RNA polymerase II from the miRNA gene [93]. These pri-miRNAs are cleaved in the cell nucleus by an enzyme called Drosha and processed into precursor miRNAs (pre-miRNAs) [94]. Pre-miRNAs are transported to the cytoplasm, where they are further processed by an enzyme called Dicer, resulting in a double-stranded mature, functional miRNA composed of approximately 22 nucleotides. Subsequently, Argonaute binds to the double-stranded mature miRNA [95]. One of the two strands, known as the guide strand, remains in Argonaute, forming the RNA-induced silencing complex, while the other strand, referred to as the passenger strand, is degraded. The guide strand within the RNA-induced silencing complex binds complementarily to the target mRNA, leading to its degradation or the inhibition of translation, thereby directly regulating the expression of the target gene [96].

Research on miRNAs has significantly expanded our understanding of gene regulation and their involvement in various diseases, including cancer, neurodegenerative disorders, and cardiovascular diseases [95,97,98]. Among these, miRNAs exhibit aberrant expression patterns and play a significant role in gene regulation, making them potential targets for diagnosis and treatment (Table 1). In particular, miRNAs influence major signaling pathways in CRC, as discussed in Section 2, resulting in changes in tumor phenotype [99]. For example, miR-590-5p was overexpressed in CRC patient tissues and cell lines and was verified to directly regulate the expression of PDCD4, thereby reducing its expression [100]. It was confirmed that the decreased miR-590-5p induced the expression of PDCD4, resulting in modulating the expression of TGF-β and SMAD2/3, genes involved in the TGF-β signaling pathway. In addition, the downregulation of miR-590-5p induced declined viability, migration, and invasion, suggesting its potential as a therapeutic target for CRC treatment. Similarly, miR-150 was downregulated in both CRC patient tissues and cell lines, exhibiting an inverse expression pattern with β-catenin [101]. Further experiments confirmed *CTNNB1*, encoding β-catenin, as a target gene of miR-150. The overexpression of miR-150 resulted in decreased cell proliferation, inhibiting CRC progression in mouse models, indicating its involvement in the Wnt signaling pathway and potential as a therapeutic target. In another study, miR-130a-3p was downregulated in CRC patient tissues and various cell lines [102]. The overexpression of miR-130a-3p led to decreased proliferation, viability, and tumor size, implicating its role in the Wnt signaling pathway by reducing the expression of its target gene, *Wnt1*. These findings suggest the therapeutic potential of targeting miR-130a-3p in CRC treatment.

In addition, numerous studies have shown that miRNAs affect CRC by regulating the expression of genes for which the signaling mechanisms involved have not yet been exactly identified. For instance, RNF187, known for its tumor-suppressive role in other cancers, was overexpressed in CRC patient tissues and cell lines, correlating negatively with poor prognosis [103]. Furthermore, miR-144-5p was decreased in patient tissues and directly regulated the expression of RNF187. Artificial knockdown of RNF187 resulted in suppressed proliferation, migration, and invasion while increasing apoptosis. This suggests that miR-144-5p regulates the expression of RNF187, impacting the progression of CRC and highlighting its potential as a biomarker for CRC diagnosis and therapy. Similarly, research on miR-1-3p suggests its potential as a novel therapeutic strategy for CRC [104]. Downregulated in CRC patient tissues and cell lines, miR-1-3p directly regulated overexpressed YWHAZ. The overexpression of miR-1-3p led to reduced proliferation and invasion, as well as the inhibition of EMT, impacting the development and progression of CRC.

**Table 1:** The functions of miRNAs with differential expression relating to the main signaling pathways implicated in CRC, as well as experimental information for validation.

miRNA Expression	miRNAs	Target Genes	Clinical Value	Impact on CRC	Function	Signaling Pathway	Human Samples	Study Model	References
UP	miR-590-5p	*PDCD4*	therapeutic target	pathogenesis	induce cell viability, migration, and invasion	TGF-β signaling pathway	30 tumor and adjacent normal tissues	in vitro	[100]
	miR-21	*VMP1*	therapeutic target	development	promote migration and invasion, while repressing autophagy and drug sensitivity	PTEN/AKT/TFEB signaling pathway	4 tumor and adjacent normal tissues	Oncomine dataset, in vitro	[105]
	miR-21-5p	*TGF-β1*	therapeutic target	NA	induce pyroptosis while inhibiting cell viability	NA	5 tumor and normal tissues	in vitro	[106]
	miR-452-5p	*PKN2*	therapeutic target	progression	enhance cell proliferation, cell cycle transition, and chemoresistance, while suppressing apoptosis	MAPK/ERK signaling pathway	87 tumor and matched para-carcinoma mucosal tissues	TCGA, in vitro, in vivo	[107]
		*DUSP6*							
	miR-106-5p	*FAT4*	NA	carcinogenic	promote proliferation, migration, invasion, and angiogenesis	NA	tumor and adjacent normal tissues	TCGA, in vitro	[108]
	miR-496	*RASSF6*	therapeutic target	progression	induce cell motility, migration, invasion, and EMT	Wnt signaling pathway	28 tumor and adjacent normal tissues	TCGA, in vitro	[109]
	miR-125b	*CFTR*	diagnosis biomarker and therapeutic target	metastasis	enhance migration, invasion, metastasis, and EMT	RhoA/ROCK signaling pathway	58 tumor and adjacent normal tissues	human tissue microarray chips, in vitro, in vivo	[110]
		*CGN*							
	miR-298	*PTEN*	therapeutic target	development	facilitate cell metabolic activity, cell cycle progression, migration, and invasion, while inhibiting apoptosis	AKT/ERK and AKT/mTOR/P70 S6K signaling pathways	100 tumors and matched adjacent normal tissues, 100 tumor and normal plasma	in vitro	[111]
	miR-429	*LATS2*	diagnosis biomarker and therapeutic target	tumorigenesis	stimulate proliferation and tumor growth	YAP/TAZ signaling pathway	21 tumor and normal tissues	in vitro, in vivo	[112]
	miR-645	*EFNA5*	therapeutic target	metastasis	enhance migration, invasion, metastasis, and tumor growth	NA	28 tumor and adjacent normal tissues	in vitro, in vivo	[113]
DOWN	miR-150	*CTNNB1*	therapeutic target	progression	inhibit cell proliferation	Wnt signaling pathway	30 tumor and adjacent normal tissues	in vitro, in vivo	[101]
	miR-130a-3p	*WNT1*	biomarker, therapeutic target	development	repress cell proliferation and tumor growth	Wnt signaling pathway	30 tumor and adjacent normal tissues	TCGA, in vitro, in vivo	[102]
	miR-144-5p	*RNF187*	diagnosis biomarker and therapeutic target	progression	suppress migration and invasion	NA	83 tumor and adjacent normal tissues	in vitro	[103]
	miR-1-3p	*YWHAZ*	therapeutic target	progression	inhibit cell proliferation and EMT	NA	20 tumor and adjacent normal tissues	GEO database, in vitro	[104]
	miR-144-3p	*BCL6*	prognosis biomarker and therapeutic target	progression	repress cell proliferation and cell cycle progression	Wnt signaling pathway	20 tumor and adjacent normal tissues	in vitro	[114]
	miR-450a-5p	*SOX2*		progression	suppress stemness, vasculature, and tumor growth	NA	90 glass-slide tumor tissues for array	in vitro, in vivo	[115]
	miR-215-5p	*CTNNBIP1*	therapeutic target	progression and metastasis	inhibit clonogenic potential, cell cycle, migration, invasion, metastasis, and tumor growth, while inducing apoptosis	Wnt signaling pathway	primary tumor, paired liver metastatic, and adjacent normal tissues	in vitro, in vivo	[116]
	miR-148b	*p55PIK*	therapeutic target	progression	suppress proliferation, tumor growth	p53 signaling pathway	10 tumor and normal tissues	in vitro, in vivo	[117]
	miR-16	*Survivin*	therapeutic target	NA	repress proliferation and induce apoptosis	p53 signaling pathway	52 tumor and adjacent normal tissues	in vitro	[118]
	miR-139-3p	*KRT80*	therapeutic target	NA	inhibit proliferation, migration, and invasion	NA	27 tumor and normal tissues	in vitro	[119]
	miR-125a-5p	*FNDC3B*	prognosis biomarker and therapeutic target	progression	reduce proliferation	PI3K/mTOR signaling pathway	36 tumor and adjacent normal tissues	in vitro	[120]
	miR-217								
	miR-654-3p	*SRC*	diagnosis and prognosis biomarker, and therapeutic target	development	repress proliferation, migration, and invasion, while promoting apoptosis	NA	103 tumor and adjacent normal tissues	in vitro, in vivo	[121]
	miR-539	*TIPE*	therapeutic target	progression	suppress proliferation and tumor growth, while enhancing ferroptosis	SAPK/JNK signaling pathway	26 tumor and adjacent tissues	in vitro, in vivo	[122]
	miR-3622a-3p	*SALL4*	therapeutic target	progression and metastasis	reduce proliferation and EMT	Wnt signaling pathway	80 tumor and adjacent normal tissues	TCGA, in vitro, in vivo	[123]

### 3.2. Competing Endogenous RNA (ceRNA) Regulation in CRC

CeRNAs refer to various RNA molecules containing MREs that compete for miRNA binding, of which lncRNAs and circRNAs are well known [124,125]. This dynamic interaction establishes the ceRNA network, where miRNAs compete for interactions with lncRNAs or circRNAs as well as mRNAs. Consequently, it is known that ceRNAs function as a miRNA sponge because they are involved in various physiological processes, such as development, by binding to miRNAs and regulating the availability and activity of miRNAs [126,127,128]. Recent studies have extensively investigated ceRNAs in numerous diseases, particularly CRC [129]. CeRNAs play a pivotal role in CRC initiation and progression by interfering with the binding between miRNAs and mRNAs, thereby serving as potential therapeutic targets or diagnostic biomarkers [130].

#### 3.2.1. LncRNA/miRNA/mRNA

LncRNAs are a class of RNA molecules longer than 200 nucleotides that do not encode proteins but play diverse roles in many biological processes, such as transcriptional control, post-transcriptional processing, and cellular organization, exerting a crucial influence on gene expression regulation [131,132,133,134,135,136]. LncRNAs are transcribed from lncRNA genes by RNA polymerase II (Pol II), synthesizing the nascent lncRNA transcript (Figure 2) [137,138,139]. Similar to mRNA, the nascent lncRNA transcript undergoes processes, including 5′ capping, splicing, and 3′ poly-A tailing, to be stabilized. Some of the produced lncRNAs function in the nucleus, while others transport to the cytoplasm for regulatory roles, where they interact with other RNA molecules, such as miRNAs [140,141]. This interaction influences cellular processes associated with cancer, particularly revealing significant impacts on CRC, suggesting their potential as therapeutic targets for CRC (Table 2) [141,142,143,144].

Accumulated studies have indicated that abnormal expression levels of lncRNAs play an important role in the development and progression of CRC acting as miRNA sponges. In particular, genes implicated in CRC-related signaling pathways, as highlighted in Section 2, are regulated through the interaction of lncRNAs and miRNAs. For example, the overexpression of CTBP1-AS2 in CRC has been identified as a miRNA sponge, influencing CRC development [145]. Increased CTBP1-AS2 activated TGF-β, thereby promoting the proliferation and metastasis of CRC cells while concurrently suppressing apoptosis. Experimental evidence further supported the notion that upregulated miR-93-5p led to a reduction in the expression of both CTBP1-AS2 and TGF-β1, establishing miR-93-5p as a common target for these two factors. Similarly, the increase in SNHG16 contributed to the proliferation of CRC cells, promoting tumor development in CRC [146]. This study revealed that the overexpressed SNHG16 interacted with miR-302-3p, influencing the expression of AKT, a target gene of miR-302-3p. Additionally, the upregulation of miR-302-3p resulted in the increased proliferation of CRC cells, providing evidence that SHNG16 acts as a sponge for miR-302-3p.

In addition to the previously mentioned genes related to important CRC pathways, lncRNAs also play a significant role in CRC by regulating the expression of genes involved in various signaling pathways. In a study, both *HIF1A*, a target gene of miR-20b-5p, and COL4A2-AS1 expression were overexpressed in CRC patient tissues and cell lines, while miR-20b-5p was decreased [147]. COL4A2-AS1 was observed to compete with *HIF1A* and acted as a miRNA sponge for miR-20b-3p. Furthermore, reducing COL4A2-AS1 expression in cells and mouse models resulted in decreased cell viability, proliferation, and aerobic glycolysis, thus inhibiting tumorigenesis. These findings suggest the potential of COL4A2-AS1 as a novel target for CRC therapy. In another study, MIR503HG was upregulated in CRC patients, with a more pronounced decrease observed in stages III-IV compared to stages I-II [148]. Its expression was downregulated in patients with lymph node metastasis compared to those without. Increased MIR503HG led to the inhibition of cell proliferation, migration, and invasion, and the induction of apoptosis. Further investigations revealed that MIR503HG inversely regulated miR-107, and PAR4 was negatively controlled by miR-107, establishing a positive relationship between PAR4 and MIR503HG. It was demonstrated that MIR503HG acts as a tumor suppressor by inhibiting CRC development and metastasis, suggesting its potential as a target for CRC treatment.

**Table 2:** The functions of lncRNAs with differential expression relating to the main signaling pathways implicated in CRC, as well as experimental information for validation.

LncRNA Expression	LncRNAs	miRNAs	Genes	Clinical Value	Impact on CRC	Function	Signaling Pathway	Human Samples	Study Model	References
UP	EGFR-AS1	miR-133b	*STAT3*	early diagnosis biomarker	development and progression	induce cell proliferation, migration, and invasion	NA	130 tumor and 30 normal tissues	in vitro	[142]
	CTBP1-AS2	miR-93-5p	*TGF-β1*	NA	progression and metastasis	promote proliferation, invasion, and metastasis, while inhibiting apoptosis	TGF-β signaling pathway	50 tumor and normal tissues	GEPIA, in vitro, in vivo	[145]
	SNHG16	miR-302a-3p	*AKT*	therapeutic target	development	enhance proliferation	AKT signaling pathway	NA	in vitro	[146]
	COL4A2-AS1	miR-20b-5p	*HIF1A*	biomarker and therapeutic target	progression	facilitate proliferation and aerobic glycolysis	NA	55 tumor and adjacent normal tissues	in vitro, in vivo	[147]
	MIR4435-2HG	miR-206	*YAP1*	prognosis biomarker and therapeutic target	metastasis	stimulate invasion, migration, EMT, metastasis, and growth	Hippo signaling pathway	90 tumor and normal tissues	in vitro, in vivo	[149]
	NEAT1	miR-205-5p	*VEGFA*	diagnosis biomarker and therapeutic target	development	induce proliferation, migration, and invasion	NA	30 tumor and adjacent normal tissues	in vitro	[150]
		miR-34a	*SIRT1*	prognosis biomarker and therapeutic target	metastasis	enhance proliferation, invasion, and tumor growth	Wnt signaling pathway	100 tumor and normal tissues	GEO database, in vitro, in vivo	[151]
	HOTAIR	miR-206	*CCL2*	therapeutic target	progression	stimulate proliferation and invasion, while inhibiting apoptosis	NA	32 tumor and normal tissues	TCGA, GEPIA, UALCAN, SurvExpress, in vitro	[152]
	HCG18	miR-1271	*MTDH*	therapeutic target	development	promote proliferation and invasion	Wnt signaling pathway	20 tumor and adjacent normal tissues	StarBase, in vitro	[153]
	RoR	miR-6833-3p	*SMC4*	NA	tumorigenesis	facilitate proliferation and viability, while inhibiting apoptosis	NA	24 tumor and normal tissues	in vitro	[154]
	SNHG8	miR-588	*ATG7*	therapeutic target	development	induce proliferation and autophagy	NA	NA	TCGA, in vitro	[155]
	MAFG-AS1	miR-149-3p	*HOXB8*	prognosis biomarker and therapeutic target	progression	stimulate proliferation, migration, and invasion	NA	30 tumor and adjacent normal tissues	in vitro, in vivo	[156]
	SNHG6	miR-181b-5p	*JAK2*	biomarker and therapeutic target	progression	enhance proliferation, while repressing apoptosis	NA	40 tumor and adjacent normal tissues	in vitro	[157]
		miR-181c-5p								
	RHPN1-AS1	miR-7-5p	*OGT*	therapeutic target	progression	promote proliferation, migration, and invasion, while suppressing apoptosis	NA	NA	in vitro, in vivo	[158]
	CASC21	miR-7-5p	*YAP1*	therapeutic target	progression	facilitate migration, invasion, and EMT, while inhibiting apoptosis	NA	NA	GEPIA, in vitro	[159]
	HOXD-AS1	miR-526b-3p	*CCND1*	NA	progression	stimulate proliferation, migration, and invasion	NA	54 tumor and adjacent normal tissues	in vitro	[160]
	RP11-757G1.5	miR-139-5p	*YAP1*	prognosis biomarker and therapeutic target	progression and metastasis	enhance proliferation, cell cycle progression, migration, invasion, tumor growth, and metastasis	NA	43 tumor and 6 adjacent normal tissues	in vitro, in vivo	[161]
	MCF2L-AS1	miR-874-3p	*CCNE1*	diagnosis and prognosis biomarkers and therapeutic target	initiation and progression	induce proliferation, migration, invasion, and EMT, while inhibiting apoptosis	NA	130 tumor and normal tissues	in vitro	[162]
	DANCR	miR-185-5p	*HMGA2*	NA	progression	promote proliferation, migration, and invasion	NA	50 tumor and adjacent normal tissues	in vitro	[163]
	RNCR3	miR-1301-3p	*AKT1*	therapeutic target	progression	facilitate proliferation and invasion, while suppressing apoptosis	NA	76 tumor and adjacent normal tissues	in vitro, in vivo	[164]
DOWN	MIR503HG	miR-107	*PAR4*	therapeutic target		suppress migration and invasion	NA	80 tumor and adjacent normal tissues	in vitro, in vivo	[148]
	LINC00485	miR-581	*EDEM1*	therapeutic target	progression and metastasis	repress proliferation, migration, invasion, tumor growth, and metastasis	NA	52 tumor and adjacent normal tissues	TCGA, GEO database, in vitro, in vivo	[165]
	DPP10-AS1	miR-127-3p	*ADCY1*	therapeutic target	progression	inhibit stemness, sphere formation, proliferation, migration, invasion, and tumor growth, while enhancing apoptosis	NA	54 rumor and adjacent normal tissues	in vitro, in vivo	[166]
	MBNL1-AS1	miR-412-3p	*MYL9*	therapeutic target	progression, occurrence	repress proliferation, invasion, migration, and tumor formation, while increasing apoptosis	NA	NA	TCGA, GEO database, in vitro, in vivo	[167]
	FENDRR	miR-18a-5p	*ING4*	NA	progression and metastasis	suppress proliferation, migration, invasion, tumor growth, and metastasis	NA	42 tumor and adjacent normal tissues	GEPIA, in vitro, in vivo	[168]
	MCM3AP-AS1	miR-19a-3p	*FOXF2*	biomarker and therapeutic target	progression	inhibit proliferation and migration	NA	53 tumor and adjacent normal tissues	GEPIA, in vitro	[169]

#### 3.2.2. CircRNA/miRNA/mRNA

CircRNAs are endogenous ncRNAs found in various species, including mammals [170,171]. These molecules exhibit a continuous circular structure, connecting the 3′ and 5′ ends through a phosphodiester bond, and notably lack both a 5′ cap or a 3′ poly-A tail [172,173]. This unique characteristic renders them considerably more stable compared to linear RNAs, providing resistance to exonuclease activity. While circRNAs are not fully understood, they are known to be produced through various synthetic mechanisms, including back-splicing, intron-pairing-driven circularization, and lariat-driven circularization (Figure 2). Back-splicing, a non-canonical splicing event, deviates from the typical splicing pathway that leads to the formation of linear mRNA from precursor mRNA (pre-mRNA) [174,175]. Here, a canonical splice site (5′-GU and 3′-AG at intron) is used, where the 5′ splice donor site downstream of the pre-mRNA connects with the 3′ splice acceptor site upstream, forming a 3′, 5′-phosphodiester bond [173,176,177]. This process results in circRNAs, as the end of the downstream exon is ligated to the beginning of the upstream exon. In intron-pairing-driven circularization, circRNAs are formed by the base pairing of reverse complementary sequences, such as Alu elements, within the flanking long introns of pre-mRNA [178,179]. The third biogenesis type, lariat-driven circularization, occurs through canonical splicing events [178,180,181]. In canonical splicing events, the intronic region of pre-mRNA is debranched, leading to the excision of the intron, and exons are ligated to generate mature mRNA. However, if the debranching process escapes, the lariat structure persists due to the 2′, 5′-phosphodiester bond between the splice donor and branch point. This failure to dismantle the lariat structure maintains the circular form, making it a precursor for circRNAs. Similar to lncRNAs, circRNAs function in the nucleus and play crucial roles in the cytoplasm. Residing in the cytoplasm and containing MRE, circRNAs function as sponges for miRNA, allowing them to act as transcriptional regulators [173,182]. Ultimately, the ability of circRNAs to indirectly regulate mRNA expression has revealed diverse biological functions. Specifically, it has been demonstrated that changes in circRNA expression in CRC impact the initiation, growth, and metastasis of CRC (Table 3) [183,184,185]. Consequently, the altered expression of circRNAs is recognized as a potential biomarker for the diagnosis or treatment of CRC [186,187].

In particular, there are studies on circRNAs that indirectly regulate the expression of genes involved in the key signaling pathways mentioned in Section 2, which play a significant role in CRC. In a study, circ_0008285 expression was downregulated in CRC patient tissues and cell lines [188]. The expression level of circ_0008285 was inversely correlated with tumor size, lymph node metastasis, and tumor–node–metastasis stage. Furthermore, the knockdown of circ_0008285 resulted in the enhanced proliferation and migration of cell lines, revealing its role as a sponge for miR-382-5p. Since miR-382-5p acts as a regulator of PTEN involved in the PI3K/AKT signaling pathway, circ_0008285 affected CRC by indirectly regulating PTEN and is suggested as a potential target for CRC treatment. In a different investigation, the downregulated circ_0009361 in both colorectal cancer patient tissues and cell lines was associated with the promotion of proliferation, EMT, migration, and invasion [189]. Conversely, the overexpression of hsa_circ_0009361 resulted in reduced tumor size and decreased metastasis. Further experiments revealed that hsa_circ_0009361 acted as a miRNA sponge by binding to miR-582, thereby regulating the expression of APC2. Consequently, hsa_circ_0009361 influences colorectal cancer by interacting with miR-582 to regulate APC2 involved in the Wnt signaling pathway.

In addition, it has been revealed that circRNAs can compete with genes unrelated to the main signaling pathways known to have a significant impact on CRC, thereby regulating miRNA. In a study, the expression of circCTNNA1 was upregulated in CRC tissues and associated with poor prognosis, especially in advanced tumor stages showing node metastasis [190]. It has been revealed that circCTNNA1 competed with FOXM1, a target gene of miR-149-5p, thus binding to miR-149-5p. The knockdown of circCTNNA1 resulted in decreased proliferation and tumor size. Therefore, circCTNNA1 plays an oncogenic role, highlighting its potential as a target for CRC therapy and diagnosis. Similarly, circ_GLG1 exhibited high expression in CRC patients, and the downregulation of circ_GLG1 led to the suppression of tumor cell viability, proliferation, migration, and invasion [191]. Circ_GLG1 was identified to act as a sponge for miR-622, influencing the expression of *KRAS*, a target gene of miR-622. This suggests that circ_GLG1 holds potential as a novel biomarker in CRC diagnosis. In another study, it was observed that circ0065378 expression was decreased in CRC tissues and cell lines [192]. Artificially increasing circ0065378 expression led to reduced proliferation, migration, and invasion of CRC cells. Circ0065378 was found to interact with miR-4701-5p, indirectly regulating the target gene *TUSC1* of miR-4701-5p, thereby influencing malignant behavior. These findings suggest that circ-PLXNB1 can serve as a novel target for the diagnosis and treatment of CRC.

**Table 3:** The functions of circRNAs with differential expression relating to the main signaling pathways implicated in CRC, as well as experimental information for validation.

CircRNA Expression	CircRNAs	miRNAs	Genes	Clinical Value	Impact on CRC	Function	Signaling Pathway	Human Samples	Study Model	References
UP	CircVAPA	miR-125a	*CREB5*	therapeutic target	progression	induce cycle progression, glycolysis, migration, and invasion	NA	42 tumor and normal tissues	in vitro	[187]
	CircCTNNA1	miR-149-5p	*FOXM1*	diagnosis biomarker and therapeutic target	progression	facilitate proliferation, DNA synthesis, migration, invasion, and tumorigenesis	NA	60 tumor and adjacent normal tissues	TCGA, in vitro, in vivo	[190]
	CircGLG1	miR-622	*KRAS*	diagnosis biomarker and therapeutic target	progression	promote proliferation, invasion, and migration	NA	40 tumor and normal tissues	in vitro	[191]
	CircSPARC	miR-485-3p	*JAK2*	diagnosis and prognosis biomarkers and therapeutic target	progression and metastasis	enhance proliferation, migration, invasion, tumor growth, and metastasis	JAK/STAT signaling pathway	84 tumor and adjacent normal tissues, 40 plasmas of patients	GEO database, in vitro, in vivo	[193]
	Circ0007142	miR-122-5p	*CDC25A*	diagnosis biomarker and therapeutic target	progression	stimulate proliferation, migration, invasion, and tumor growth	NA	31 tumor and adjacent normal tissues	in vitro, in vivo	[194]
		miR-455-5p	*SGK1*	therapeutic target	progression	induce proliferation, migration, invasion, and tumor growth while repressing apoptosis	NA	45 tumor and para-carcinoma tissues	in vitro, in vivo	[195]
	Circ0000467	miR-382-5p	*EN2*	therapeutic target	development	enhance proliferation, migration, invasion, and EMT	NA	69 tumor and adjacent normal tissues	GEO database, in vitro	[196]
	Circ0001178	miR-382	*ZEB1*	therapeutic target	metastasis	stimulate migration, invasion, EMT, and metastasis	NA	102 tumor tissues	in vitro, in vivo	[197]
		miR-587								
		miR-616								
	Circ0053277	miR-2467-3p	*MMP14*	therapeutic target	development and progression	promote proliferation, migration, and EMT	NA	3 tumor and normal tissues	in vitro	[198]
	Circ0060745	miR-4736	*CSE1L*	therapeutic target	NA	accelerate proliferation, migration, and invasion	NA	28 tumor and para-tumor tissues	in vitro	[199]
	Circ102209	miR-761	*RIN1*	therapeutic target	progression	enhance proliferation, cell cycle progression, migration, invasion, EMT, and tumor growth, while suppressing apoptosis	NA	56 tumor and para-carcinoma tissues	human circRNA array v2, in vitro, in vivo	[200]
	Circ100146	miR-149	*HMGA2*	early diagnosis biomarker and therapeutic target	progression and metastasis	facilitate proliferation, migration, invasion, tumor growth, and metastasis, while inhibiting apoptosis	NA	58 tumor and normal tissues	GEO database, in vitro, in vivo	[201]
	Circ0004277	miR-512-5p	*PTMA*	NA	progression	induce proliferation and tumor growth, while inhibiting apoptosis	NA	50 tumor and para-carcinoma tissues	GEO database, in vitro, in vivo	[202]
	CircFARSA	miR-330-5p	*LASP1*	therapeutic target	progression	promote proliferation, migration, invasion, and tumor growth	NA	40 tumor and adjacent normal tissues	in vitro, in vivo	[203]
	Circ000166	miR-326		diagnosis biomarker and therapeutic target	progression	stimulate proliferation, while repressing apoptosis	NA	40 tumor and adjacent normal tissues	GEO database, in vitro	[204]
	CircPRMT5	miR-377	*E2F3*	therapeutic target	progression	facilitate proliferation and tumor growth	NA	30 tumor and adjacent normal tissues	in vitro, in vivo	[205]
	CircERBIN	miR-125a-5p	*4EBP-1*	therapeutic target	progression	enhance proliferation, migration, invasion, tumor growth, angiogenesis, and metastasis	NA	59 tumor and adjacent normal tissues	in vitro, in vivo	[206]
		miR-138-5p								
	CircMAT2B	miR-610	*E2F1*	therapeutic target	progression	induce proliferation	NA	70 tumor and adjacent normal tissues	in vitro	[207]
	Circ0084615	miR-599	*DNMT3A*	biomarker and therapeutic target	progression and metastasis	promote proliferation, migration, invasion, and metastasis	NA	50 tumor and adjacent normal tissues	GEO database, in vitro, in vivo	[208]
	CircDENND4C	miR-760	*GLUT1*	diagnosis biomarker and therapeutic target	progression	enhance proliferation, migration, glycolysis, and tumor growth	NA	tumor and normal tissues	sequencing, in vitro, in vivo	[209]
	CircUBAP2	miR-582-5p	*FOXO1*	biomarker and therapeutic target	progression and metastasis	stimulate migration, invasion, proliferation, autophagy, tumor growth, and metastasis	NA	3 tumor and normal tissues	in vitro, in vivo	[210]
	Circ0089153	miR-198	*SENP1*	therapeutic target	progression	facilitate proliferation, sphere formation, tube formation, and tumor growth, while suppressing apoptosis	NA	50 tumor and adjacent noncancerous tissues	in vitro, in vivo	[211]
	CircNOX4	miR-485-5p	*CKS1B*	NA	progression	induce proliferation, migration, invasion, glycolysis, and tumor growth	NA	46 tumor and adjacent normal tissues	in vitro, in vivo	[212]
	CircHERC4	miR-556-5p	*CTBP2*	prognosis biomarker and therapeutic target	progression and metastasis	promote proliferation, migration, invasion, tumor growth, and metastasis	Notch signaling pathway	120 tumor and adjacent normal tissues	sequencing, in vitro, in vivo	[213]
	Circ0000372	miR-495	*IL6*	prognosis biomarker and therapeutic target	progression	enhance proliferation, migration, invasion, and tumor growth	JAK/STAT signaling pathway	60 tumor and adjacent normal tissues	in vitro, in vivo	[214]
	CircLDLR	miR-30a-3p	*SOAT1*	biomarker and therapeutic target	progression and metastasis	stimulate proliferation, DNA synthesis, cholesterol, migration, invasion, tumor growth, and metastasis	NA	80 tumor and 15 normal tissues	in vitro, in vivo	[215]
DOWN	Circ0008285	miR-382-5p	*PTEN*	therapeutic target	progression	repress proliferation and migration	PI3K/AKT signaling pathway	56 tumor and noncancerous colorectal mucosa tissues	in vitro	[188]
	Circ_0009361	miR-582	*APC2*	therapeutic target	progression and metastasis	inhibit proliferation, EMT, migration, invasion, tumor growth, and metastasis	Wnt signaling pathway	30 tumor and paracancerous tissues	microarray, in vitro, in vivo	[189]
	Circ0065378	miR-4701-5p	*TUSC1*	diagnosis biomarker and therapeutic target	progression	suppress proliferation, migration, invasion, EMT, and tumor growth	NA	25 tumor and adjacent normal tissues	sequencing, in vitro, in vivo	[192]
	CircCUL2	miR-208a-3p	*PPP6C*	diagnosis biomarker and therapeutic target	progression	repress proliferation, apoptosis, and tumor growth, while increasing apoptosis	NA	30 tumor and adjacent normal tissues	microarray, in vitro, in vivo	[216]
	CircSMARCA5	miR-93-5p	*ARID4B*	biomarker	progression	inhibit proliferation, migration, invasion, and tumor growth	NA	tumor and adjacent tissues	in vitro, in vivo	[217]
	Circ0003266	miR-503-5p	*PDCD4*	therapeutic target	progression	suppress proliferation, migration, and invasion, while accelerating apoptosis	NA	46 tumor and paracancerous tissues	GEO database, in vitro	[218]

## 4. Conclusions

**Figure 3:**
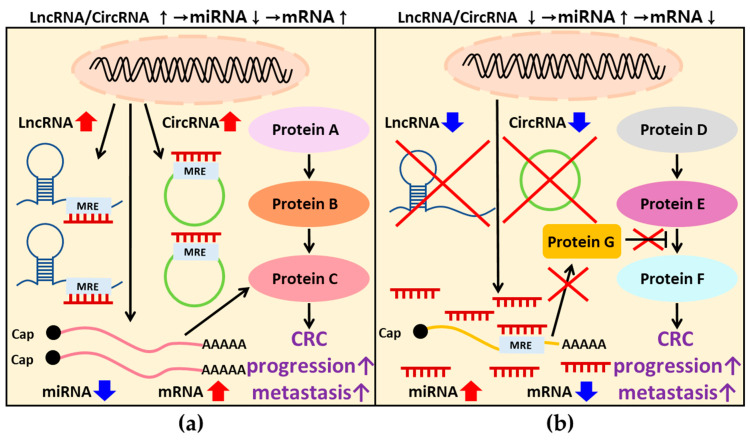
The regulatory mechanisms of ceRNA/miRNA/mRNA to enhance CRC progression or metastasis: (**a**) Upregulated ceRNA binds to miRNA, interfering with the binding between miRNA and target mRNA. This interference results in increased mRNA expression, promoting CRC progression or metastasis. (**b**) Downregulated ceRNA induces an increase in miRNA expression, leading to the binding to mRNA, which acts as an inhibitor of signaling pathways. Consequently, this interaction induces a decrease in mRNA expression, promoting the progression or metastasis of CRC. ceRNA: competing endogenous RNA; miRNA: microRNA; mRNA: messenger RNA; CRC: colorectal cancer.

According to numerous studies and KEGG pathway analyses, the key signaling pathways in CRC are the Wnt, PI3K/AKT/mTOR, MAPK, TGF-β, and p53 signaling pathways. Genes involved in each signaling pathway have been shown to influence behaviors of CRC cells, such as proliferation, migration, and invasion, ultimately impacting the progression and metastasis of CRC. Additionally, extensive research has been conducted on ncRNAs in CRC, as they are known regulators of genes. Overexpressed miRNAs have been found to suppress the expression of tumor suppressor genes, while downregulated miRNAs increased the expression of oncogenes, exerting negative effects on CRC. Furthermore, the discovery of ceRNAs has drawn attention to their indirect regulation of gene expression through the interaction between ceRNAs and miRNAs, thus influencing CRC (Figure 3). However, there are several limitations in the current research. Firstly, some studies solely rely on either in vitro or in vivo experiments, lacking validation in patient samples. Secondly, certain studies are exclusively based on in vitro experiments, lacking confirmation of reproducibility in vivo. Thirdly, the functional roles of genes regulated by ncRNAs remain ambiguous. As discussed in Section 2, although significant signaling pathways in CRC have been identified, Section 3 shows a deficiency in research on ncRNAs regulating genes involved in major signaling pathways. Instead, much research has been performed on gene regulation whose mechanisms have not yet been revealed. Therefore, for the studied ncRNAs to have clinical value, in-depth studies are needed on the regulation of genes involved in key signaling pathways or the mechanisms of genes whose exact functions remain unidentified. As these studies accumulate, it is expected that it will be possible to discover meaningful biomarkers or suitable treatment targets in CRC.

## Data Availability

Not applicable.
